# Emulating the Electrical Activity of the Neuron Using a Silicon Oxide RRAM Cell

**DOI:** 10.3389/fnins.2016.00057

**Published:** 2016-02-23

**Authors:** Adnan Mehonic, Anthony J. Kenyon

**Affiliations:** Department of Electronic and Electrical Engineering, University College LondonLondon, UK

**Keywords:** resistive switching, neuronal dynamics, Hodgkin-Huxley, leaky integrate-and-fire, memristor

## Abstract

In recent years, formidable effort has been devoted to exploring the potential of Resistive RAM (RRAM) devices to model key features of biological synapses. This is done to strengthen the link between neuro-computing architectures and neuroscience, bearing in mind the extremely low power consumption and immense parallelism of biological systems. Here we demonstrate the feasibility of using the RRAM cell to go further and to model aspects of the electrical activity of the neuron. We focus on the specific operational procedures required for the generation of controlled voltage transients, which resemble spike-like responses. Further, we demonstrate that RRAM devices are capable of integrating input current pulses over time to produce thresholded voltage transients. We show that the frequency of the output transients can be controlled by the input signal, and we relate recent models of the redox-based nanoionic resistive memory cell to two common neuronal models, the Hodgkin-Huxley (HH) conductance model and the leaky integrate-and-fire model. We employ a simplified circuit model to phenomenologically describe voltage transient generation.

## Introduction

Software models, supported by digital architecture, are convenient means to study the quantitative behavior of biological neural networks in the field of computational neuroscience. However, they cannot simulate large-scale neural systems in real time. Existing hardware, based on conventional digital logic, cannot support software that mimics detailed brain activities at a realistic scale, even with huge power consumption. Hence, artificial hardware neural systems, designed using the principles of biological neural structures, are now being developed (Indiveri, 2000; Le Masson et al., 2002; Vogelstein et al., 2008; Mitra et al., 2009). These systems are often called “neuromorphic” (Mead, 1990; Indiveri et al., 2011).

Nanodevices in which an electrical stimulus modifies electrical resistance hold great potential for a wide range of applications, the most obvious being non-volatile memories. Of such technologies, Resistive Random Access Memories (RRAMs; Waser and Aono, 2007), often classed as examples of the two-terminal elements known as memristors (Chua, 1971), are being developed as alternatives to existing memory technologies (Torrezan et al., 2011; Chen et al., 2012; Mehonic et al., 2012a). However, these devices have potential applications beyond memory, as their resistance can in some cases be semi-continuously varied, rather then being limited to binary or discrete multi-state values. Such analog variation of resistance provides a useful model of key features of the biological synapse, and RRAMs as synapses in neuromorphic circuits promise high density and efficient processing. There have been numerous recent reports of synaptic behavior such as spike timing dependent plasticity in RRAMs (Jo et al., 2010; Indiveri et al., 2013; Yu et al., 2013; Saïghi et al., 2015). However, when it comes to modeling neuronal behavior, a hybrid approach is employed in which a RRAM/memristor models a biological synapse while CMOS circuits model neuronal dynamics. By modeling both the synapse and the neuronal electrophysiological conductance/voltage response in one device, hardware neural networks can be much simpler than existing hybrid analog/digital CMOS silicon neurons. This is the goal of the work we report here.

Here we demonstrate the feasibility of using the RRAM cell to model aspects of the electrical activity of the neuron; more specifically, the generation of voltage transients that may begin to model an action potential—neuronal spiking. Further, we demonstrate the integration capability of the device—a crucial aspect of neuronal dynamics. We discuss the operational procedures required to generate spike-like responses; we compare these spikes with those observed in biological neurons, and we relate recent models of redox-based nanoionic resistive memory cells to the conductance-based models of the neural membrane [the leaky integrate-and-fire model and the Hodgkin-Huxley (HH) model]. Although a detailed description of the physical mechanism responsible for spiking is outside the scope of this paper, we use a simple RC circuit model, similar to the one used in the leaky integrate-and-fire model, to discuss spike generation.

## Materials and methods

Our test devices are SiO_x_ MIM (metal-insulator-metal) RRAM structures consisting of 37 nm-thick SiO_x_ layers (*x* = 1.3) sandwiched between 100 nm-thick TiN electrodes, defined by standard photolithography. Individual device sizes range from 400 × 400 to 5 × 5 μm. More details of fabrication and characterization are given elsewhere (Mehonic et al., 2015). Electrical measurements employ a Keithley Instruments 4200-SCS semiconductor parameter analyser and a Signatone probe station with 10 μm tip diameter tungsten probes. MATLAB Simulink is used for the circuit analysis.

## Results

More details of the resistance switching of our devices can be found in our previous study (Mehonic et al., 2015). Suffice it to say that devices require an initial abrupt electroforming step to move them from a highly insulating pristine state to a low resistance state (LRS). Subsequent resetting steps put them into a high resistance state intermediate between the LRS and pristine states. The pristine state is never recovered. Switching occurs by the formation of conductive filaments (Buckwell et al., 2015) of oxygen vacancies bridging the oxide. Devices can be cycled any times between the high and LRSs by applying the appropriate voltage or current stimuli. Transitions between states are typically fast—nanoseconds or shorter. Under unipolar operation, in which transitions from HRS to LRS and from LRS to HRS occur for the same polarity voltage stimulus, a current compliance limit is used during the HRS to LRS transition to prevent destructive breakdown of the conductive filament due to runaway Joule heating. For the opposite transition the current compliance is removed, and thermally-assisted diffusion of oxygen resets the device to the HRS.

We define two distinct classes of resistance switching: memory switching and threshold switching. The former is characterized by its non-volatility—devices remain in a specific resistance state until a stimulus causes a transition. Depending on the past history of the device, a given read voltage can result in one of two or more different currents, with the device cycled between the different states by voltage or current pulses. This is the switching mode that enables digital or multi-level operation. Threshold switching, on the other hand, is the mode in which a device is in one resistance state for low read voltages or currents, and in a different state for higher. This is a volatile system in which the measured resistance is a function of the read voltage or current.

First we examine the metastable device states that enable a fast voltage response. We explore two ways to achieve this. The first one considers typical memory switching. The second one considers threshold switching.

### Generation of controlled voltage transients (voltage spikes) using memory switching

**Figure 1:**
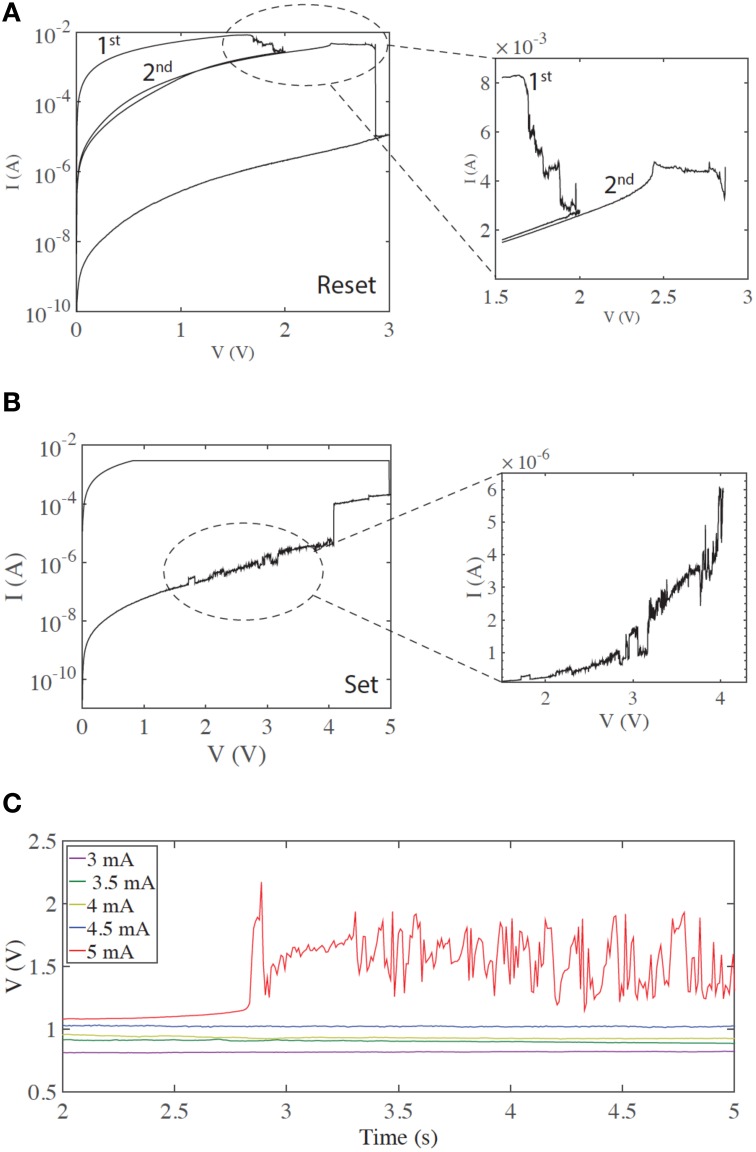
**Regions of current instabilities in I/V sweeps of SiO_*x*_ RRAM cells during non-volatile memory switching. (A)** A gradual reset process, before an abrupt transition to the HRS. The zoomed region highlights the region of instability. **(B)** Current instability before an abrupt set process. **(C)** Voltage response with a constant current input, demonstrating the threshold effect of the voltage transients (spikes). In this case, no voltage transients or spikes are observed until a current of 5 mA is applied to the device.

First we examine typical unipolar memory (non-volatile) switching. We obtain this type of switching by setting a higher current compliance—typically around 3 mA for our devices. The zoomed-in current-voltage curves in Figures 1A,B demonstrate regions of rich electrical dynamics, which are either around the transitions between the two stable states (HRS and LRS)—or regions shortly before these thresholds. Resetting (the transition from LRS to HRS) is typically gradual (Figure 1A), in contrast to the abrupt electroforming and setting processes. By stopping the voltage sweep at different points along this process, multi-level switching can be obtained. The end of the reset process is typically a more abrupt transition to the HRS (Mehonic et al., 2015). In the case shown, three distinct resistance states are obtained by stopping the first sweep at 2 V and the second at 3 V. Such multi-level switching is typically used to model a biological synapse. Many current spikes typically follow the overall increase of resistance.

Setting (the HRS to LRS transition) is typically an abrupt single process, although more than one level can often be observed and multi-level switching achieved (Mehonic et al., 2012b). In many cases current spikes or instabilities are observed shortly before the threshold voltage (Figure 1B).

We tested the generation of voltage transients (resembling voltage spikes) by applying a constant current bias to our devices and measuring the resultant voltage response. This is similar to intracellular recording from neurons using the current clamp method, tracking the generation of the action potentials. In the following text we assume that a voltage spike is an abrupt voltage increase followed by abrupt voltage decrease. More specifically, whenever the voltage increase and subsequent decrease is greater than the standard deviation of the whole signal, and is shorter than 200 ms (typically three data points), we consider that to be a voltage spike. This is quite a relaxed definition of a voltage spike and should not be confused with the more defined stereotypical shape of the action potential generated in a biological neuron. We examined the stable, typical memory switching shown in Figures 1A,B, now applying a constant current and monitoring device voltage. Figure 1C demonstrates the resulting threshold voltage spiking/instability. Below a threshold current (here 5 mA), the voltage response is constant with no spikes. However, once the input current is above threshold significant spiking is observed. This usually occurs after some time, indicating integration of the input signal over time. Such behavior is equivalent to the neuronal generation of action potentials above a threshold input. Voltage spiking continues for a long period of time (typically >5 s) and is sometimes followed by transition to an intermediate metastable state, from which spiking resumes either spontaneously or after further increasing the input current. If current is reduced below threshold, spiking stops and a constant voltage response is recovered. A subsequent increase of input current above threshold triggers spiking again. The threshold current is usually finely defined and is approximately the same as the reset current. As the reset current is defined by current compliance during electroforming/setting (Russo et al., 2009), the threshold may be electrically tailored.

**Figure 2:**
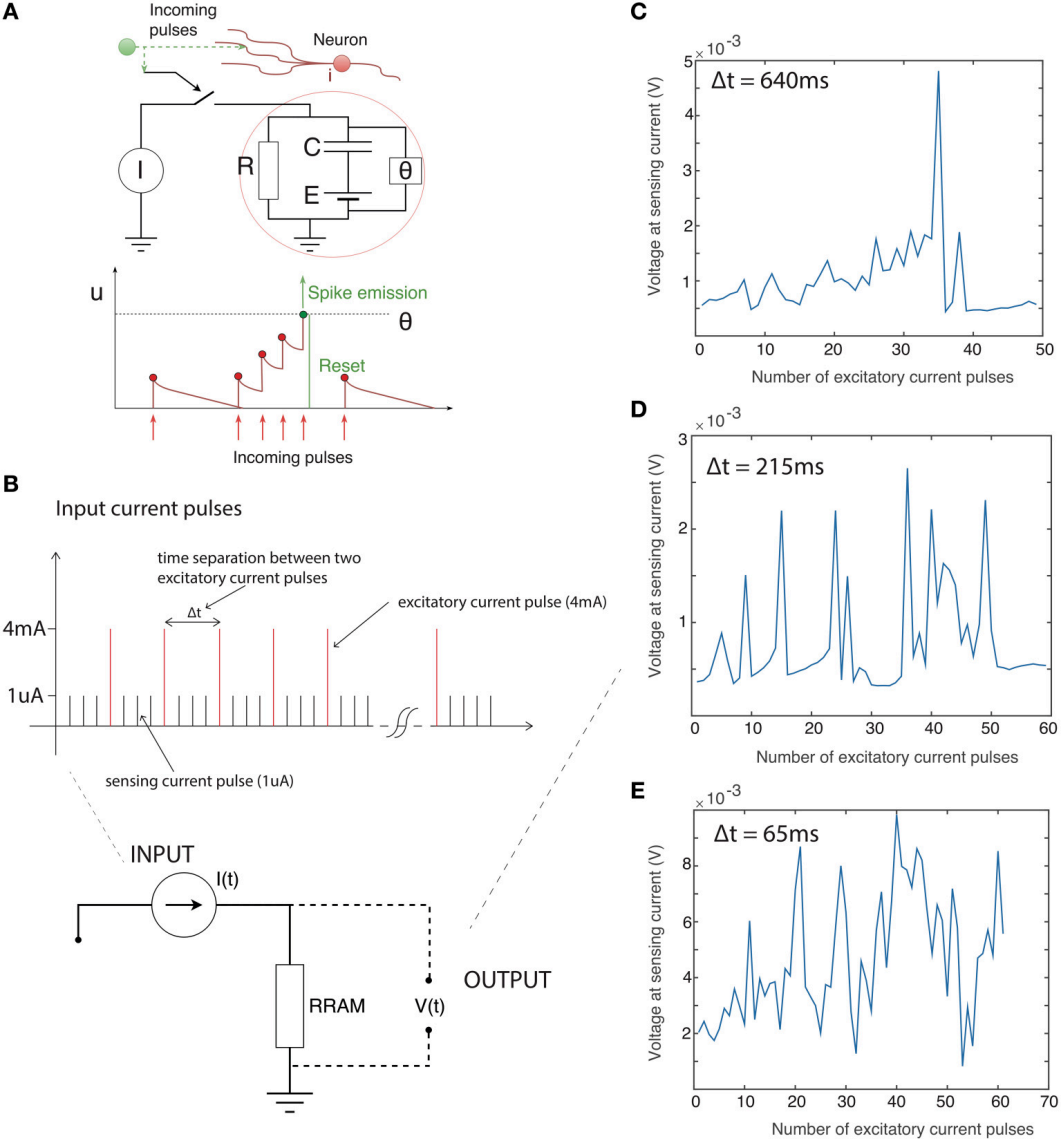
**(A)** Basic representation of leaky integrate-and-fire neuronal model. Upper: schematic of model. Theta defines the voltage threshold for spiking. Lower: illustration of integration of input current pulses to generate voltage spike. X-axis is time, y axis is neuronal potential. **(B)** Time sequence of input to device: Train of excitatory current pulses (4 mA) separated by sensing current pulses (1 uA). Output of device: Voltage response measured only with the sensing 1 uA current pulses immediately after the excitatory 4 mA current pulse with the time separated of **(C)** 640 ms **(D)** 215 ms **(E)** 65 ms. The number of pulses required to be integrated decreases as the inter-pulse interval becomes shorter.

We explored the integration capability of our devices by applying a train of current pulses instead of a constant current bias. For the particular device reported here the threshold current level was around 4 mA (slightly over the 3 mA current compliance), thus we applied 4 mA excitatory current pulses (pulse width approximately 65 ms) followed by a train of 1 uA sensing pulses to track the voltage change across the device. One microampere is well below the threshold level, and hence these pulses are negligible compared to the much larger 4 mA excitatory pulses. Summing only the number of 4 mA pulses can approximate integration of the input current signal. We varied the time separation between the excitatory pulses to examine the capacity for current-time integration. Results are presented in Figure 2. Figure 2A shows the main concept of integration in the leaky integrate-and-fire model. A train of closely-spaced current pulses builds up a potential across the neural membrane until, at a specified threshold, theta, the neuron generates a voltage transient. If the separation between input current pulses is large there is a significant discharge of a membrane capacitor between the two pulses thus it takes more pulses for a voltage spike to be generated. Conversely, if pulses are more frequent the voltage spike will be generated after a fewer input pulses. We use the same analogy here, though the voltage across the device is now tracked by 1 uA sensing current pulses. Figure 2C shows the voltage across the device (sensed with a 1 uA current pulse) after every 4 mA excitatory pulse. The time separation between excitatory pulses is around 640 ms. A gradual build up of the voltage across the device is apparent before the voltage spike after around 35 excitatory pulses. The voltage spike is generated quicker (after fewer excitatory pulses) if the pulse separation is decreased. Figures 2D,E show the voltage after every excitatory pulse when the pulses are separated by 215 and 65 ms, respectively. This clearly shows the relation between the time separation between the pulses and generation of the voltage spike. This behavior is phenomenologically similar to charging and discharging of the membrane capacitor in the leaky integrate-and-fire model.

### Generation of controlled voltage transients (voltage spikes) using threshold switching

**Figure 3:**
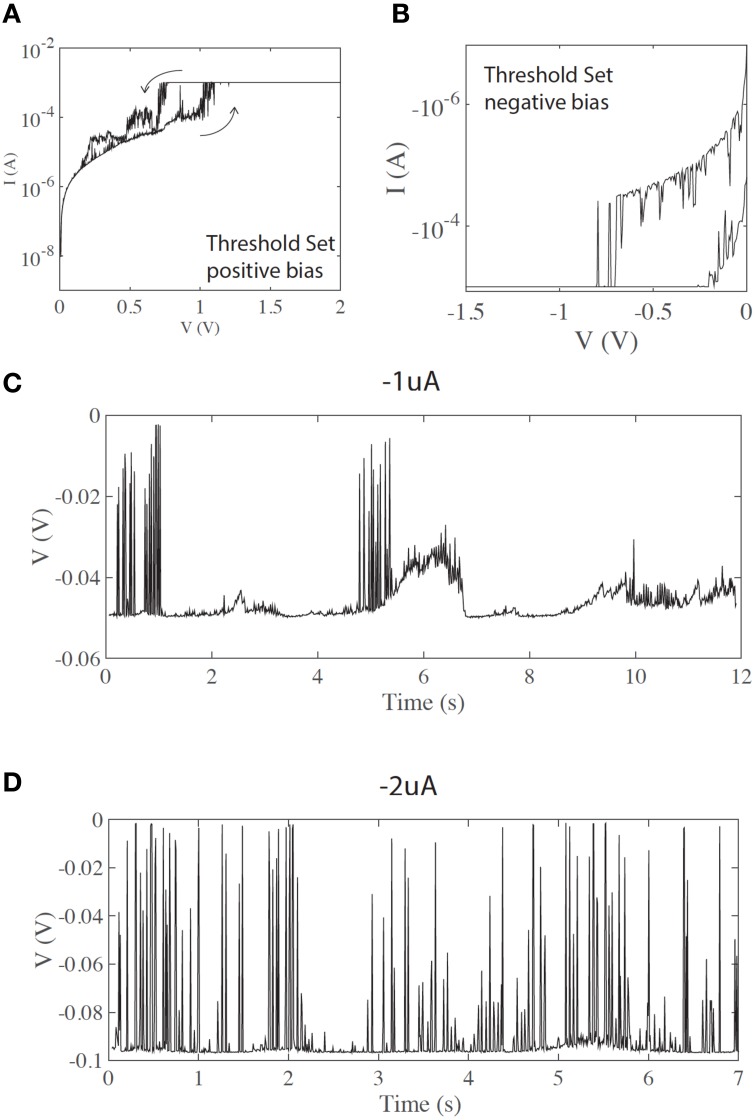
**(A,B)** threshold (volatile) switching in positive and negative bias, respectively. **(C)** Short voltage spikes are observed even at lower current inputs (negative current input). **(D)** Same as **(C)** under increased current input, showing a higher count of voltage transients.

In some cases devices exhibit volatile, threshold-like resistance switching, which can be initiated by using lower current compliance during the electroforming and set process. It is known that the diameter of the conductive filament produced during the electroforming step is controlled by current compliance (Ielmini, 2011; Ielmini et al., 2011). Thinner filaments, produced with lower current compliance, are less stable, and exhibit higher volatility, as seen in Figures 3A,B. Both states (LRS and HRS) exhibit large current instabilities.

In the case of volatile/threshold resistance switching (Figures 3A,B), fast spiking is observed even for lower current inputs. Figures 3C,D show spiking for negative currents of −1 and −2 uA, respectively. Although not fully controllable, the input current can affect the pattern of spikes. Figure 3C shows a chattering-like firing pattern similar to that often seen in biological neurons. Figure 3D shows a different firing pattern, similar to fast spiking. Although the threshold current is less finely defined than in the case of memory (non-volatile) switching, a strong correlation with the input current is evident.

**Figure 4:**
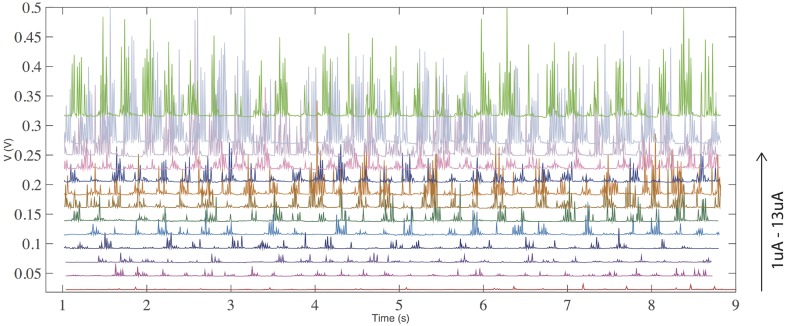
**(color online) Voltage response with a constant current input for threshold (volatile) switching**. The frequency of spiking/firing is increased with an increase of the input current.

Figure 4 demonstrates the effect of increasing input current from 1 to 13 uA. Less prominent firing is observed at lower currents, while the firing frequency is increased by raising the current. This is a signature of a neuronal response.

**Figure 5:**
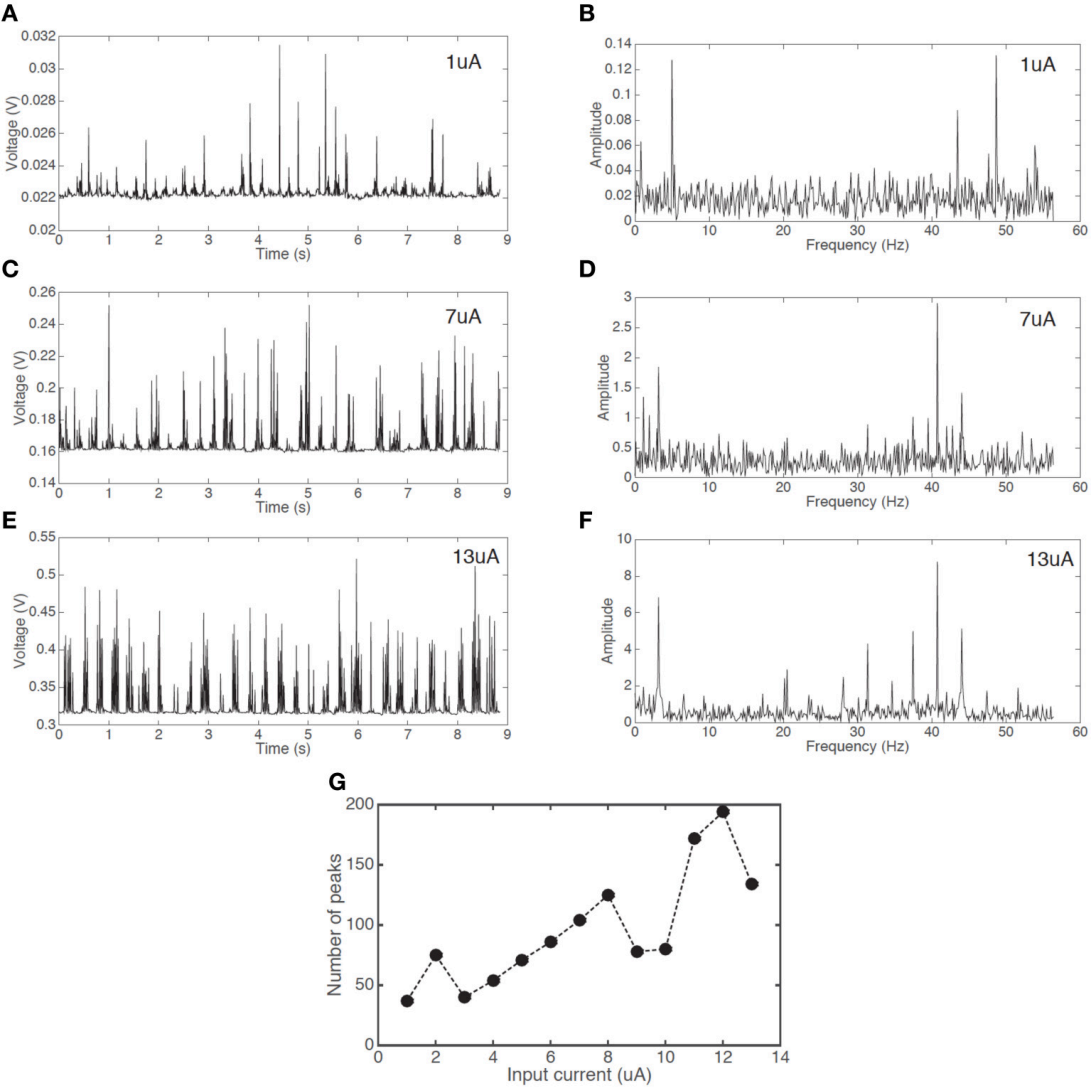
**Voltage responses with a constant current input and the corresponding Fourier transforms**. Spiking signal with an input current of **(A)** 1 uA **(C)** 7 uA **(E)** 13 uA, and Fourier transform signal with the input current of **(B)** 1 uA **(D)** 7 uA **(F)** 13 uA. **(G)** The increase in the number of peaks in an interval of 8.8 s with increasing input current.

Firing events are not fully random. There is a clear pattern of a fast firing sequence followed by a refractory period of no firing. To further study this behavior we analyzed the dynamics of the firing pattern. Figures 5A,C,E show the firing patterns of three different input currents (1, 7, and 13 uA respectively). Figures 5B,D,F show the corresponding Fourier transform of the signals. It is apparent that for all three signals there are two dominant frequencies (a first peak in region 4–5 Hz and a second peak in region of 40–50 Hz). This behavior is similar for all signals shown in Figure 4. Figure 5G demonstrates an increase in the number of peaks (proportional to an average firing frequency) with increased input current.

Regardless of the input current, the overall spiking pattern, resembling a chattering pattern, stays unchanged.

Generation of voltage transients (spikes) using threshold switching is less controlled than when using memory switching, although some level of control (firing frequency) is still retained. However, this approach has certain advantages. Voltage spikes are typically more pronounced and the overall operational energy is significantly lower then for the first approach using memory switching (currents of a few microAmps are sufficient to generate voltage spikes). On the other hand, on-volatile operation provides very good control of the threshold levels as well as integration of the input signal.

## Discussion

### Comparison of the extended memristor model of the ReRam system with the hudgkin-huxley and leaky-and-integrate neuronal models

**Figure 6:**
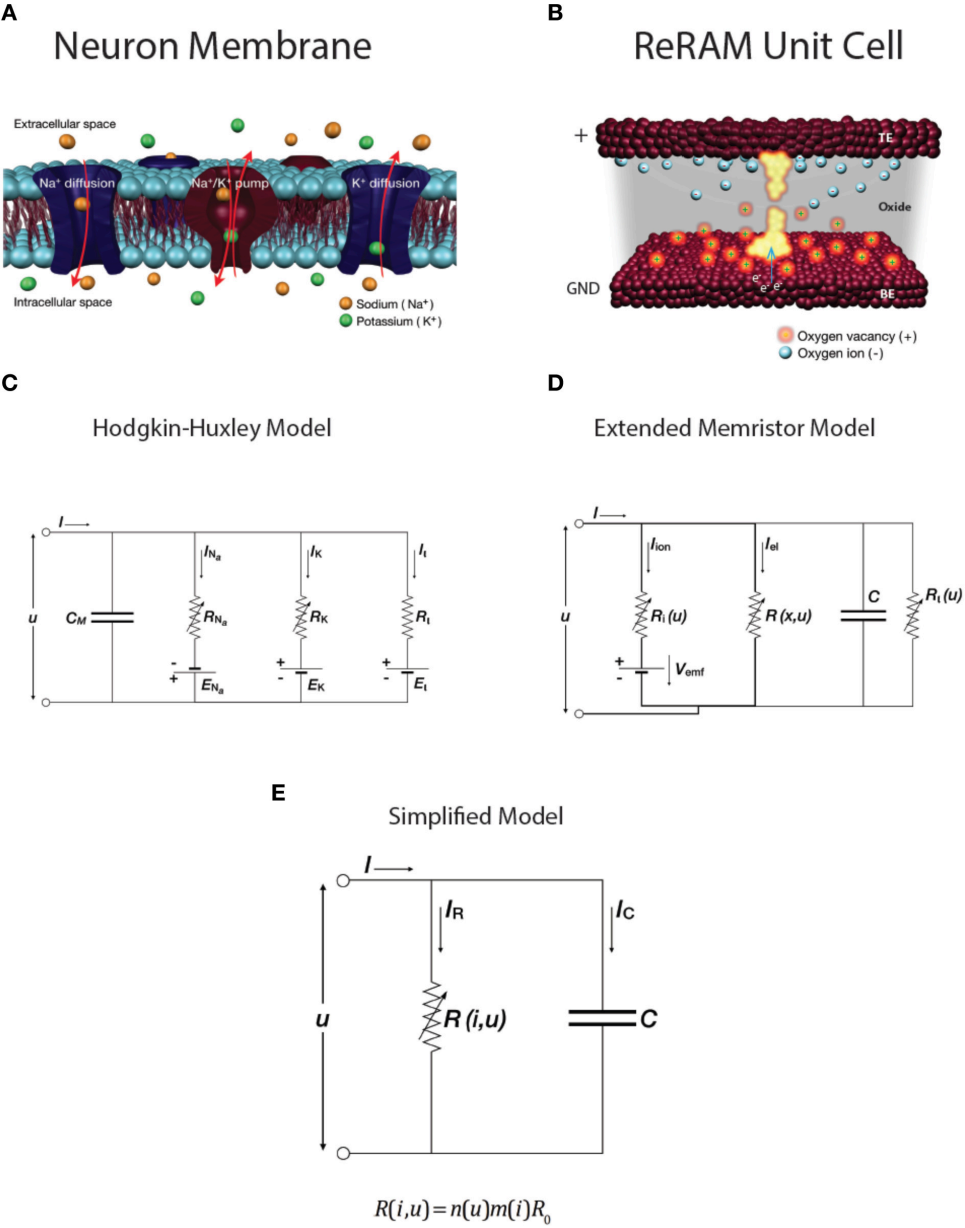
**(color online) Schematics of (A) a neuron cell membrane and (B) a ReRAM unit cell**. Equivalent circuits of **(C)** Hodgkin-Huxley conductance-based model of neuron membrane and **(D)** extended memristive element. **(E)** Simplified RC model with variable resistance R.

A detailed description of the switching mechanism can be found in our previous work, though we note here that it falls within the description of redox-based nanoionic resistive memories (Waser et al., 2012; Mehonic and Kenyon, 2015). Here we will discuss the similarities and differences between the biological system described by the HH model and leaky integrate-and-fire model, the extended memristor model of ReRAM system, and our device. Schematic representations of the two systems are shown in Figures 6A,B. We first compare the latest redox-based nanoionic model of resistance switching (Valov et al., 2013) with the conduction-based Hodgkin-Huxley model of the neuron (Hodgkin and Huxley, 1952). The easiest way to analyse the similarities is to compare the two equivalent electric circuits. The nanoionic model takes into account the non-equilibrium states inside the memory cell and the generation of an internal electromotive force (*V*_*emf*_) by the movement of ions during electrical biasing. This requires an expansion of memristor theory to include a nanobattery; the resultant equivalent circuit is shown in Figure 6D. This is the extended memristance model.

The Hodgkin-Huxley model provides an electrical description of the generation of the action potential. A set of differential equations describes the conductance of the neuron membrane, with the equivalent circuit shown in Figure 6C. It assumes two ionic channels (usually sodium and potassium) and one nonspecific leakage channel, as well as corresponding ion pumps. Changes in the membrane potential and in the conductivity of the ion channels generate the action potential. The model is summarized by Equation (1). The ion currents on the right-hand side are sodium, Na^+^, potassium, K^+^, and the leakage current. When the ion channels are fully open they have maximum conductances *g*_*Na*_, *g*_*K*_, respectively. The dynamics of the variable conductivity are defined by the gating variables *n, m* and *h*, which model ion channel opening. A generalized gating variable *x* is defined by a differential equation (Equation 2), with both steady state gating variable *x*_0_ and time constant τ_*x*_ dependent on voltage *u*. Since there is a build up of the Nernst potential across the membrane for every ionic species, there are additional battery elements. These are modeled by *E*_*N*__*a*_, *E*_*K*_, and *E*_*L*_.

(1) $$\begin{array}{l} \sum_{k}I_{k}=g_{Na}m^{3}h\left(u-E_{Na}\right)+g_{K}n^{4}\left(u-E_{K}\right)+g_{L}\left(u-E_{L}\right) \end{array}$$

(2) $$\begin{array}{l} \frac{dx}{dt}=-\frac{x-x_{0}\left(u\right)}{\tau_{x}\left(u\right)} \end{array}$$

The circuit representation of the HH model is very similar to that of the Extended Memristor Model (EMM; Figures 6C,D). Both include a capacitance in parallel with one or more variable resistors and internal emf sources. Unsurprisingly, the EMM can be described by a similar set of equations to those of the HH model, including contributions from ionic and electrical currents and a built-in emf (Equation 3).

(3) $$\begin{array}{l} I=I_{ion}\left(V_{emf},u\right)+I_{el}\left(x,u\right)=G\left(x,u\right)\times \left(u-t_{ion}V_{emf}\right) \end{array}$$

With ionic current *I*_*ion*_ and electronic current *I*_*el*_. The former is defined by the nanobattery, *V*_*emf*_. The latter is controlled by state-dependent *x*. *G* is the conductance, *u* is applied voltage, and *t*_*ion*_ is the transference number (the total ionic transfer number). More details and a derivation of the model can be found in Valov et al. (2013).

Importantly for our discussion this V_emf_ is very small in the case of Valence Change Memory systems such as our SiO_x_ devices (Valov et al., 2013). This contribution is further reduced when the device is in the LRS. Similarly, the ionic resistance, R_i_, is very large compared to the electronic resistance R. We may therefore make a useful simplification to the equivalent circuit model, shown in Figure 6E, which includes a single variable resistance.

### Phenomenological modeling of the dynamics of a non-volatile SiO_x_ RRAM device

To analyse the dynamics of our SiO_x_ RRAM system, more specifically to phenomenologically describe the generation of voltage transients, and to make comparison with neuronal dynamics, we consider the simplified model in Figure 6E. It is worth noting that a simple RC circuit is used in the leaky integrate-and-fire neuron models to integrate the input signal. In these models, the RC circuit does not generate any voltage spikes, but it provides a measure of voltage increase across the membrane (membrane capacitor) and when the threshold voltage is reached a separate external circuit is used to generate a voltage spike. After this voltage spike is generated the voltage across the RC circuit is reset. In contrast, in our model we do not use additional circuit elements to generate spikes; instead we examine the effect of the dynamically variable resistance *R*. Resistance is a general function of both the applied voltage and the passing current. This is similar to the HH model, in which ion channel conductance is dynamically controlled by the voltage across the neural membrane. Consequently, to model voltage spike generation in our device (using non-volatile memory switching) we use some elements of both the HH model (voltage controlled resistance *R*) and the leaky integrate-and-fire neuronal model (*RC* equivalent circuit).

Although resistance transitions are controlled both by electric field and associated Joule heating, in the case of unipolar switches the set process is triggered predominantly by the electric field (voltage), while Joule heating (current) triggers the reset. To a good approximation this means that, above a certain value, current breaks the filament and increases the overall resistance, while the voltage restores the filament and reduces the resistance. This is modeled phenomenologically by two variable coefficients: the setting coefficient *n(u*), and the resetting coefficient *m(i*), which are phenomenologically similar to the gating coefficients in the HH model. R_0_ is the previous steady state resistance. The two coefficients, *n(u)* and *m(i)*, do not have a deeper physical meaning, but they do qualitatively describe the resistance increase with current increase and the resistance decrease with voltage increase above the threshold.

(4) $$\begin{array}{l} R\left(i,u\right)={n\left(u\right)m\left(i\right)R}_{0} \end{array}$$

**Figure 7:**
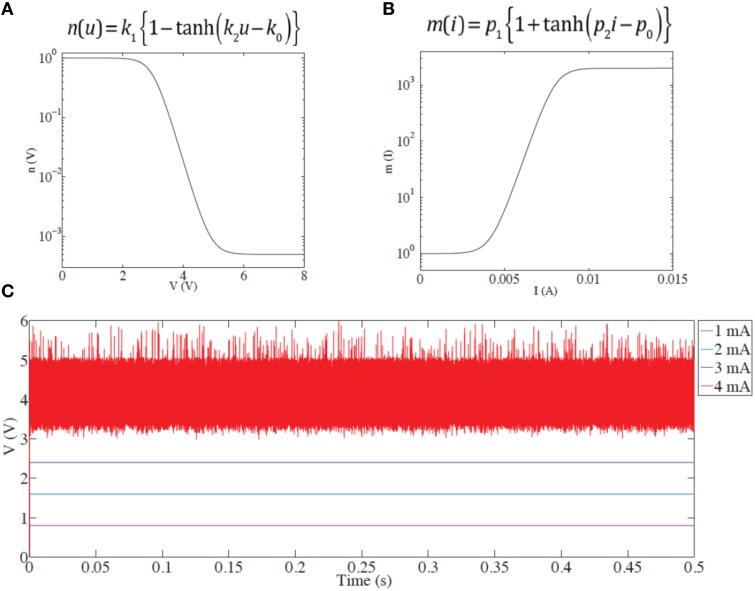
**(A)** Setting coefficient dependence on applied voltage. **(B)** Resetting coefficient dependence on current. In both cases, the functional forms phenomenologically describe changes in resistance in response to applied voltages and currents. The resistance change is immediate; time evolution is not taken into account in this model. **(C)** Demonstration of the instability/spiking threshold. Below threshold (*i* < 4 mA), no spikes are seen. Above threshold, multiple transients result from the competition between set and reset processes, governed by *n(u)* and *m(i)* acting in opposition. The spikes are of qualitative nature and do not describe timing.

We use this circuit model to probe the origin of voltage spiking. The input current is kept constant, and the dynamics of the device voltage are observed. For the sake of simplicity and convenience we choose two continuous functions of the following form to model the coefficients *n(u)* and *m(i)*:

(5) $$\begin{array}{l} n\left(u\right)=k_{1}\left\{1-tanh\left(k_{2}u-u_{0}\right)\right\} \end{array}$$

(6) $$\begin{array}{l} m\left(i\right)=p_{1}\left\{1+tanh\left(p_{2}i-i_{0}\right)\right\} \end{array}$$

where *k*_1_*, k*_2_*, p*_1_*, p*_2_ are unitless fitting parameters and *u*_0_*, i*_0_ are fitting parameters related to the thresholds of voltage and current governing setting and resetting, respectively. The functional shapes of the two coefficients are shown in Figures 7A,B.

Results from the above model are shown in Figure 7C. Voltage transients are observed only when the input current reaches a level of 4 mA. In our previous work (Mehonic et al., 2012a) we have discussed competition between the set and reset processes during constant voltage bias. Similar dynamics occur under current bias. If the initial state is the LRS and the input current is high enough to trigger a reset, this will drive the device toward the HRS. Consequently, device resistance will increase, as will the voltage drop across the device. For a constant current, the voltage will increase enough to trigger the set process, putting the device back to LRS and the whole process starts again. This competition between set and reset processes, generates voltage transients.

We note that in our model we do not assume any time dependence of the two coefficients, *n(u)* and *m(i)*. Equations (5) and (6) do not include any time-dependent dynamics. Furthermore, the equations are of zero-order (changes of *n(u)* and *m(i)*, and resistance are assumed to be instantaneous). This means that the model cannot provide frequency or shape analysis of the voltage responses. Instead, the aim of the model is to phenomenologically describe voltage transients and the threshold effect. In most cases the generated transients resemble a noise-like signal (a consequence of zero order dynamics) and are likely a function of simulation step size. There is therefore no correlation between the firing frequency of the experimental result in Figure 1C and model results in Figure 7C. To include shape and the frequency analysis, coefficients *n(u)* and *m(i)* should be modeled by similar differential equations to those used for the gating coefficients *x(u)* in the HH model taking into account non-zero order dynamics and the time dependency. However, the exact relation between the resistance change and applied voltage/current in RRAM systems is not yet fully established and is outside the scope of this manuscript. Nevertheless, our model clearly demonstrates the threshold effect and generation of voltage instability, without considering time evolution.

The whole discussion above considers only memory switching. Volatile/threshold, less-stable switching provides rapid resistance variations without a finely defined threshold. We suspect that rapid resistance variations are the effect of trapping/detrapping processes or random telegraph noise (RTN) affected by the redistribution of oxygen vacancies, as discussed in Balatti et al. (2014), Choi et al. (2014). The rate of movement of oxygen vacancies is increased by increasing the current input. Consequently, we observe in volatile systems that the firing frequency is also increased—a typical neuronal response.

## Conclusion

To summarize, we have demonstrated the feasibility of using the SiO_x_ RRAM cell to model aspects of the voltage spiking activity of a biological neuron. This is a different approach from conventional synaptic modeling using RRAM devices. We elaborate the specific metastable device states required for the generation of voltage spiking, and demonstrate a dynamic voltage response to a constant input current and to a current pulse train. We discuss observation of threshold spiking as well as an increase of firing frequency with increased input current. We demonstrate the integration capability of our device. We compare the model of redox-based nanoionic resistive memory to the Hodgkin-Huxley neuron model and the leaky integrate-and-fire model. We use circuit simulations to further explain the voltage response. This study could provide a novel way of using RRAM devices in neuromorphic systems beyond the already-demonstrated capability to model a functional synapse.

## Author contributions

AM conceived the study, performed the measurements, and wrote the initial draft of the paper. AK oversaw the project and revised the manuscript.

### Conflict of interest statement

The authors declare that the research was conducted in the absence of any commercial or financial relationships that could be construed as a potential conflict of interest.
